# Role of mammography screening as a predictor of survival in postmenopausal breast cancer patients

**DOI:** 10.1038/sj.bjc.6602895

**Published:** 2005-12-06

**Authors:** J Anttinen, H Kautiainen, T Kuopio

**Affiliations:** 1 Department of Pathology, Jyväskylä Central Hospital, Keskussairaalantie 19, 40620 Jyväskylä, Finland; 2 Medcare Foundation, Torikatu 4, 44100 Äänekoski, Finland

**Keywords:** breast cancer survival, breast cancer screening, screening as independent prognostic factor

## Abstract

We examined the effect of population-based screening programme on tumour characteristics by comparing carcinomas diagnosed during the prescreening (*N*=341) and screening (*N*=552) periods. We identified screen detected (*N*=224), interval (*N*=99) and clinical cancer (*N*=229) cases. Median tumour size and proportion of axillary lymph node negative cases were 1.5 cm and 65% in the screen detected group, 2.0 cm and 44% in cases found outside the screening, and 3.2 cm and 41% in the cases from the prescreening period. Survival of the breast cancer patients was 66% (95% CI, 60–71%) in the prescreening era group and 73% (95% CI, 66–78%) in the screening era group after 10 years of follow-up. In the screening era group the survival of the screen detected cases was 86% (95% CI, 80–90%) and that of the clinical cancer cases 73% (95% CI, 66–78%) after 10 years. In multivariate analysis the risk of breast cancer death was not significantly different between the prescreening and screening periods (HR 0.82; 95% CI 0.59–1.12, *P*=0.21). Detection by screening was not an independent prognostic factor in multivariate analysis (HR 0.75; CI 95% 0.50–1.12; *P*=0.17).

Mammography service screening programmes have been introduced in more than 20 countries (Shapiro *et al*, 1998). Several guidelines and different expert groups recommend mammography screening examinations (IARC, 2002) despite the contradicting opinions questioning the positive effect of the screening (Gotzsche and Olsen, 2000). Recent evaluations have shown that service screening may be even more effective than screening in population-based trials (Duffy *et al*, 2002; Paci *et al*, 2002; Gorini *et al*, 2004). Information of actual participation to screening, data of incident cancers and detailed information of tumour characteristics have provided more precise information of breast cancer prognosis in the screened and not-screened patients (Ernst *et al*, 2004). Comparison of incident-based breast cancer mortality before and after beginning of screening has shown a significant mortality reduction (Tabar *et al*, 2003; Gorini *et al*, 2004).

It has been shown that a shift to smaller tumour size has occurred after introduction of mammography screening (Kricker *et al*, 1999; Tabar *et al*, 2003). The studies reporting the significance of mammography screening as an independent prognosticator of survival are contradictory. Screening as a mode of cancer detection was not an independent factor in a study by Klemi *et al* (2003). In contrast to this finding, a recent multicentre study reported that screening itself had an independent role in prognostication of distant recurrences of breast cancer (Joensuu *et al*, 2004). Gill *et al* (2004) also found that detection by screening was a powerful independent prognosticator.

There are only few works combining data of individual screening history, survival and hospital patient records. In this work, we present a population-based breast cancer material from a Finnish health care district and compare eras before service screening (1977–1986) and during screening (1987–1997). Analyses based on incident breast cancers of women aged 50–69 years focus in finding differences in survival between cancer groups with different modes of detection.

## MATERIAL AND METHODS

### Patients

Health Care District of Central Finland is a defined geographical and administrative area in Central Finland around the city of Jyväskylä. The average population of the district is about 260 000 and the number of women aged 50–69 years is about 28 500. In this study, we identified two groups of patients aged 50–69 years, one represented a prescreening era (years 1977–1986) and the other represented an era during a service screening (years 1987–1997, screening era group). According to the files of Finnish Cancer Registry and patient files of Health Care District the numbers of new breast cancer cases were 341 in the prescreening era group and 552 in the screening era group.

Most of the patients were operated in two hospitals of the area: Jyväskylä Central Hospital in Jyväskylä and Jokilaakso Hospital in Jämsä. Surgical and oncologic data and pathology reports were collected from patient files in 759 (85%) cases. Patient records were no longer available in 36 (4%) cases and 98 (11%) women were treated outside the Health Care District. Only invasive cancers were included in the study.

Survival data of the patients was collected from the files of Finnish Cancer Registry and Governmental Statistics Finland. Both registers utilise Finnish national social security code, which enables precise person identification. Survival data and detailed information of the causes of death were available at individual patient level in all cases. Follow-up was closed on June 2003. Median follow-up time was 12.5 years (range, 0.1–26.4 years) in the prescreening era group and 8.0 years (range, 0.1–16.4 years) in the screening era group.

In most cases the largest tumour diameter was recorded from pathology reports. If histological diameter was not available the tumour diameter was extracted from surgical files. Mammography reports of tumour size were not used. Tumour diameter was missing in 89 (10%) cases, 61 (18%) of these belonged to the prescreening era group and 28 (8%) to the screening era group.

The lymph node status was unknown in 134 cases (15%). Pathology reports of the prescreening era did not state the total number of investigated or affected lymph nodes. The axillary lymph node status was also classified only as positive or negative in 71 cases in the screening era group. The axillary dissection was omitted in 20 cases treated in Jyväskylä Central Hospital.

Staging was performed according to the TNM system of the International Union against Cancer (Sobin and Witekind, 2002).

### Screening

Population-based breast cancer service screening started in Central Finland in 1987. Women aged 50–69 were invited by a personal letter to the screening. Two-view mammography was performed and read by two specially trained radiologists every 2 years according to a detailed programme. The programme was recommended by the National Board of Health (Hakama *et al*, 1991). First screening round was completed in 1991.

Women invited to screening were identified for the present study from the registers of Cancer Society of Central Finland, which actually organised the screening. During the first years of screening (1987–1990) no computer-based databases were available. However, data of all positive screening results was registered and was available for this study. Computerised data of attendance and results of all screening examinations were recorded from the beginning of 1991 onwards. During the screening era (1987–1997) 30 251 women were invited to screening and 22 499 attended. Attendance rate was 79% at the age-group 50–59 years and 58% at the group 60–69 years. In the whole material, the attendance rate was 71%. In the screening era group, the proportion of women at the age of 60–69 years was lower compared to the prescreening era, because of low attendance rate (58%) in this age group. Altogether 59 817 screening examinations were made. There were 2.5 screening visits in average per woman. Annual call-back rate was between 1.6 and 4.8%.

Altogether 224 incident breast cancers were found at service screening (screen-detected cases) during the years 1987–97. Interval cancers were identified by comparing the files of Finnish Cancer Registry and the screening register of Cancer Society of Central Finland. Breast cancer was classified as an interval cancer if a new incident cancer occurred after negative screening during the following 2 years. Altogether 99 interval cancers were identified.

There were 169 breast cancer patients in the screening era group who never attended the screening examination before their breast cancer diagnosis (never attenders). If the cancer diagnosis was made more than 2 years after the latest screening examination the case was classified as a partial attender. This group consisted of 60 women. The groups of never attenders and partial attenders together were classified as clinical cancers (*N*=229).

## STATISTICAL METHODS

The results were expressed as median and interquartile range (IQR). Statistical comparison was made by using Mann–Whitney *U*-test. Measures with a discrete distribution are expressed as counts (%) and analysed by *χ*^2^ or Fischer–Freeman–Halton test. Kaplan–Meier curves were used to illustrate information on the cumulative proportions of survival groups tested by using log-rank methods. The prognostic factors predicting the duration of the survival time were analysed using univariate and multivariate proportional hazard regression models, called Cox's regression models.

## RESULTS

### Tumour characteristics

There was a significant change to a more favourable stage distribution during the study period from the prescreening era to the screening era. The proportion of stage I cases increased from 14 to 36% and the proportion of stage III cases decreased from 15 to 4%. The number of small tumours (⩽2 cm, T1) was clearly larger in the screening era group (54%) than in the prescreening (21%) era group (Table 1). Median tumour diameter was 1.8 cm in the screening era group (IQR 1.2, 2.5) and 3.2 cm (IQR 2.0, 4.0) in the prescreening era group (*P*<0.001).

There were significant differences in stage and tumour characteristics also in the screening era group. The proportions of stage I cases and small tumours (T1) were clearly larger in the screen-detected group than in the group of clinical cancers (Table 2). Median tumour diameter was 1.5 cm (IQR 1.0, 2.0) in the screen-detected group and 2.0 cm (IQR 1.4, 3.0) in the clinical and interval cancer groups (*P*<0.001).

### Screening

Screening found 224 (41%) cases of the total number of 552 invasive breast cancers in the screening era group. The proportion of the screen-detected cancers was 50% in the age group of 50–59 years and 27% in the group of 60–69 years. Interval cancers represented 18% of all the carcinomas and 31% of the cancers in the patients who participated the screening examination. The proportion of interval cancers was 1.7/10 000 screening examinations.

### Survival

**Figure 1:**
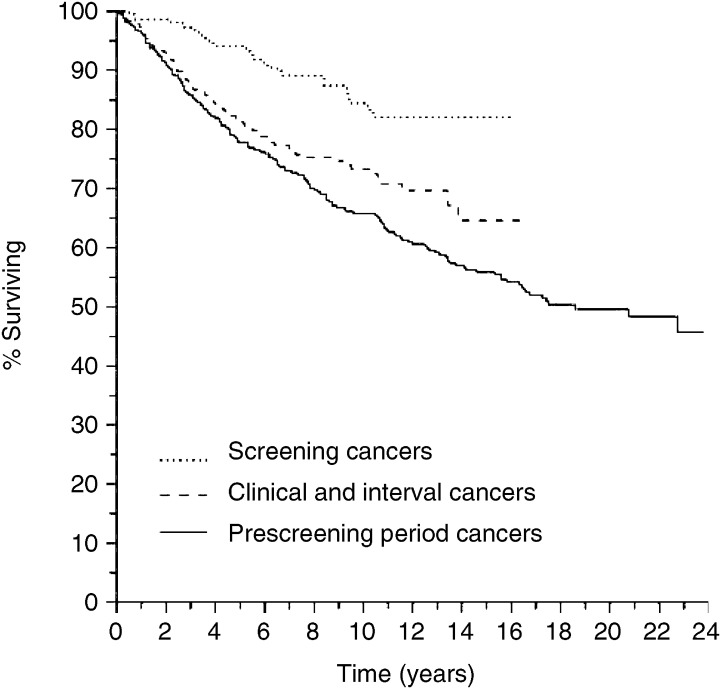
Breast cancer survival of patients with screen detected cancers (*N*=224), clinical and interval cancers (*N*=328), and prescreening period cancers (*N*=341).

**Figure 2:**
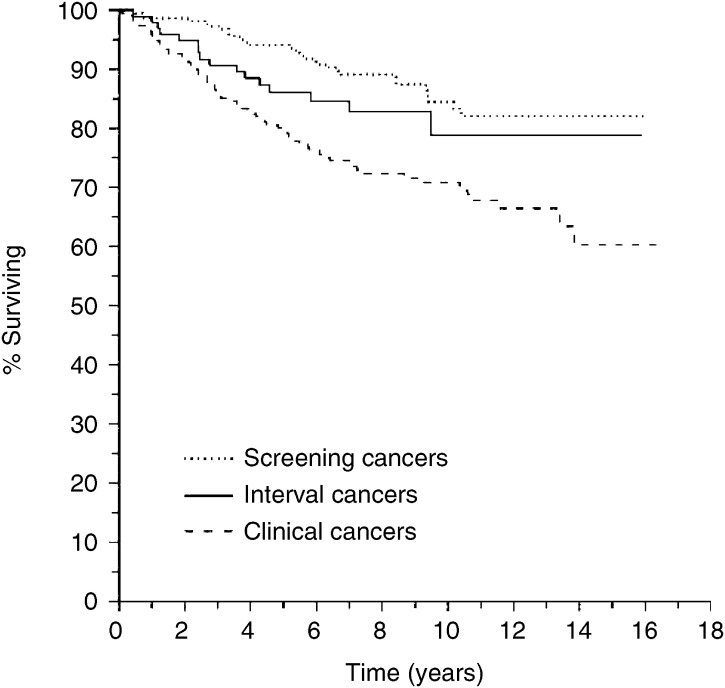
Breast cancer survival of screening period patients with screen detected cancers (*N*=224), interval cancers (*N*=99), and clinical cancers (*N*=229).

Survival of the breast cancer patients was 66% (95% CI, 60–71%) in the prescreening era group and 73% (95%CI, 66–78%) in the screening era group after 10 years of follow-up. After 5 years there was no significant difference in survival between the prescreening era cases and the clinical and interval cancer cases of the screening era period (Figure 1). In the screening era groups there was a significant (*P*<0.001) difference in survival between the screen detected (86%; 95% CI, 80–90%), interval (79%; 95% CI, 66–88%) and the clinical cancer cases (73%; 95% CI, 66–78%) after 10 years. Corresponding age adjusted significance was *P*=0.0036 (Figure 2).

When comparing crude hazard ratios of interval and screen detected cancers to clinical cancers the risk of interval cancers was of borderline significance (*P*=0.06; HR 0.58, CI 95%, 0.34–1.02) and the risk of screen-detected cancers was significantly lower (*P*<0.001; HR 0.41, CI 95%, 0.27–0.62). The hazard ratio of interval cancers did not differ significantly from screen-detected cancers (*P*=0.22; HR 1.47, CI 95%, 0.80–2.70) after adjustment by age. In multivariate analysis the risks of interval (*P*=0.69; HR 0.88, CI 95%, 0.48–1.62) and screen-detected cancers (*P*=0.32; HR 0.77, CI 95%, 0.46–1.29) were not lower when compared to clinical cancers after adjustment by age, stage, size and node status.

There was no significant difference in the risk of breast cancer death (*P*=0.21; HR 0.82, CI 95%, 0.59–1.12) between the screening era and the prescreening era groups after adjustment for tumour stage, diameter and lymph node status (Table 3). Tumour detection by screening had no independent prognostic value after corresponding adjustment (*P*=0.17; HR 0.75, CI 95% 0.50–1.12).

## DISCUSSION

In this study, we collected a population-based breast cancer material of 50–69 years old women from periods before and after onset of mammography service screening. We found that stage, tumour size and axillary lymph node status all predicted a considerably more favourable prognosis in the screening era group than in the cases diagnosed before the onset of screening. The proportion of stage III cancers reduced from 15% in the prescreening era to 4% in the screening era cases. As expected, we also found that screening detected smaller tumours with more favourable prognosis compared to the clinically detected cancers of the same era (Fracheboud *et al*, 2004). In the screen-detected group, half of the cases were stage I tumours and the proportion of stage III cases was almost nil. These findings are well in line with earlier studies showing that there will be a reduction of advanced tumour stages and increase of more favourable low stage tumours after several years of ongoing screening (McCann *et al*, 1998).

Our findings could not confirm the results of two recent studies reporting that screening itself as a mode of detection has an independent prognostic role (Gill *et al*, 2004; Joensuu *et al*, 2004). In agreement with our study Klemi *et al* (2003) reported earlier that they did not find any association between favourable prognosis and screening examination itself. These discrepancies may result from different set-ups of the studies. Our work and the study of Klemi *et al* (2003) are based on a material from a single screening centre which actually carried out the screening and maintained its own screening register and patient files. In a retrospective multicentre material of Joensuu *et al* (2004) there was a marked difference between hospital and screening register records. Only 70% of the breast cancer patients treated in the participating hospitals could be identified in the screening records. Multicentre materials may also contain larger measurement variation than data collected from a single institution.

False negative screening results and incidence of interval cancers have an important influence on the success of breast cancer screening programmes. Identification and classification of the interval cases also have an effect on estimation of survival differences between different patient groups. We paid special attention to finding all the women who got a breast cancer diagnosis within 2 years after a negative screening examination. The proportion of interval cancers (31% of the cancers in the group that participated screening) in the present work is at the same level as in many previous works (Peeters *et al*, 1989; Everington *et al*, 1999; Ernst *et al*, 2004). This indicates that the quality of the present screening programme is at an acceptable level.

In our material, the survival of interval cancers was in-between the clinical and the screen-detected cancers. Earlier studies have shown that prognosis of the interval cancers may not differ from the clinically detected cases (Brekelmans *et al* 1995; Burrell *et al*, 1996) or it is intermediate to the screen detected and the clinically detected cancer groups (Groenendijk *et al*, 2003). Several studies have shown that interval cancers form a group of tumours with aggressive characteristics (Porter *et al*, 1999; Gilliland *et al*, 2000). Besides differences in stage, size and axillary lymph node status there are other biological differences between the screen-detected, interval and clinically detected cancers, which may influence the outcome of the patients. Her-2/neu oncogene amplification (Anttinen *et al*, 2003) and high-proliferative activity (Crosier *et al*, 1999; Groenendijk *et al*, 2003) are among characteristics that are much more frequent in the interval cancers than in the screen-detected tumours.

While the adjusted effect of screening epoch on survival was not significant, it is suggestive – it might be significant if the tumour series was larger. On the other hand, it is worth making the point that in a larger tumour series, size and node status could be adjusted in more detail and this might further attenuate the effect of screening on survival in a multivariate analysis. Statistically significant differences of survival in the screening era groups disappeared after multivariate analyses.

We conclude that, as expected, breast cancer stage, tumour size and axillary lymph node status predict more favourable prognosis for the screening era patients than for the patients diagnosed before beginning of the screening mammography. These traditional prognostic markers are biologically justified and clinically clearly associated with patient outcome. In the present material with a long follow-up, we were not able to repeat the recent unexpected but intriguing finding that detection of cancer by mammography has independent favourable significance.

## Figures and Tables

**Table 1:** Characteristics of the prescreening (1977–1986) and screening era (1987–1997) breast cancers patients (*N*=893)

	**Period**		
**Characteristic**	**1977–1986 *N*=341**	**1987–1997 *N*=552**	***P*-value**
*Age (years)*			0.066
50–59, *n* (%)	185 (54)	334 (60)	
60–69, *n* (%)	156 (46)	218 (40)	
			
Stage, *n* (%)			<0.001
I	46 (14)	198 (36)	
II	157 (46)	274 (50)	
III	51 (15)	20 (4)	
Unknown	87 (25)	59 (10)	
			
*Extend of tumour (T), n (%)*			<0.001
T1	70 (21)	299 (54)	
T2	154 (45)	201 (36)	
T3	56 (16)	21 (4)	
T4	0 (0)	5 (1)	
			
Tx	61 (18)	26 (5)	
			
*Pathologic lymph node status (pN), n (%)*			<0.001
pN−	140 (41)	289 (52)	
pN+	124 (36)	206 (38)	
pX (unknown)	77 (23)	57 (10)	

**Table 2:** Characteristics of the screening era (1987–1997) breast cancer patients (*N*=552)

	**Mode of detection**			
**Characteristic**	**Screening *N*=224**	**Interval *N*=99**	**Clinical *N*=229**	***P*-value**
*Age (years)*				
50–59, *n* (%)	166 (74)	74 (75)	94 (41)	<0.001
60–69, *n* (%)	58 (26)	25 (25)	135 (59)	
				
*Stage, n (%)*				<0.001
I	111 (50)	30 (30)	57 (25)	
II	91 (40)	59 (60)	124 (54)	
III	1 (<1)	4 (4)	16 (7)	
Unknown	21 (9)	6 (6)	32 (14)	
				
*Extend of tumour (T), n (%)*				<0.001
T1	150 (67)	50 (51)	99 (44)	
T2	67 (30)	42 (42)	92 (40)	
T3	2 (<1)	3 (3)	16 (7)	
T4	0 (0)	2 (2)	3 (1)	
Tx (unknown)	5 (2)	2 (2)	19 (8)	
				
*Number of axillary metastatic lymph nodes pN*_*0–3*_ *, n(%)*				<0.001
pN_0_ (0 nodes)	145(65)	47(48)	97(42)	
pN_1_ (1–3 nodes)	37(16)	31(31)	47(21)	
pN_2_ (4–9 nodes)	16(7)	12(12)	32(14)	
pN_3_ (>10 nodes)	2(1)	3(3)	10(4)	
pN_x_ (number of nodes unknown)	24(11)	6(6)	43(19)	

**Table 3:** Cox multivariate survival analysis of the breast cancer patients

**Characteristic**	**Hazard ratio (95% CI)**	***P*-value**
Age (per year increased)	1.01 (0.98–1.04)	0.50
		
*Period*		
Prescreening (1977–1986)	Indicator	
Screening (1987–1997)	0.82 (0.59–1.12)	0.21
		
*Tumour stage*		
I	Indicator	
II	1.72 (1.13–2.64)	0.012
III	1.82 (0.81–4.09)	0.14
X (unknown)	3.26 (1.50–7.10)	0.003
		
Tumour diameter, cm	1.20 (1.06–1.37)	0.005
		
*Pathologic lymph node status (pN)*		
pN−	Indicator	
pN+	2.62 (1.91–3.61)	<0.001
pX (unknown)	2.07 (1.16–3.68)	0.013

## References

[bib1] Anttinen J, Kuopio T, Nykänen M, Torkkeli H, Saari U, Juhola M (2003) Her-2/Neu oncogene amplification and protein over-expression in interval and screen detected breast Cancers. Anticancer Res 23: 4213–4421 14666627

[bib2] Brekelmans CT, Peeters PH, Deurenberg JJ, Collette HJ (1995) Survival in interval breast cancer in the DOM screening programme. Eur J Cancer 31A: 1830–1835 8541108 10.1016/0959-8049(95)00324-c

[bib3] Burrell HC, Sibbering DM, Wilson ARM, Pinder SE, Evans AJ, Lindsey JY, Elston CW, Ellis IO, Blamey RW, Robertson JFR (1996) Screening interval breast cancers: mammographic features and prognostic factors. Radiology 199: 811–817 8638010 10.1148/radiology.199.3.8638010

[bib4] Crosier M, Scott D, Wilson RG, Griffith CDM, May FEB, Wesley BE (1999) Differences in Ki-67 and c-erbB2 expression between screen detected and true interval breast cancers. Clin Cancer Res 5: 2682–2688 10537329

[bib5] Duffy SW, Tabar L, Chen H-H, Holmqvist M, Yen MF, Abdsalah S, Epstein B, Frodis E, Ljungberg E, Hedborg-Melander C, Sundbom A, Tholin M, Wiege M, Akerlund A, Wu HM, Tung TS, Chiu YH, Chiu CP, Huang CC, Smith RA, Rosen M, Stenbeck M, Holmberg M (2002) The impact of organised mammography service screening on breast cancer mortality in seven Swedish counties. A collaborative evaluation. Cancer 95: 458–469 12209737 10.1002/cncr.10765

[bib6] Ernst MF, Voogd AC, Coebergh JW, Roukeme JA (2004) Breast carcinoma diagnosis, treatment and prognosis before and after the introduction of mass mammographic screening. Cancer 100: 1337–1344 15042665 10.1002/cncr.20139

[bib7] Everington D, Gilbert FJ, Tyack C, Warner J (1999) The Scottish screening programme's experience of monitoring interval cancers. J Med Screen 6: 21–27 10321366 10.1136/jms.6.1.21

[bib8] Fracheboud J, Otto SJ, vanDijk JAAM, Broeders MJM, Verbeek ALM, deKoonig HJ (2004) Decreasing rates of advanced breast cancer due to mammography screening in The Netherlands. Br J Cancer 91: 861–867 15292936 10.1038/sj.bjc.6602075 PMC2409867

[bib9] Gill PG, Farshid G, Luke CG, Roder DM (2004) Detection by screening mammography is a powerful independent predictor of survival in women diagnosed with breast cancer. The Breast 13: 15–22 14759711 10.1016/S0960-9776(03)00169-3

[bib10] Gilliland FD, Joste N, Stauber PM, Hunt WC, Rosenberg R, Redlich G, Key CR (2000) Biologic characteristics of interval and screen detected carcinomas. J Natl Cancer Inst 92: 743–749 10793111 10.1093/jnci/92.9.743

[bib11] Gorini G, Zappa M, Miccinesi G, Paci E, Costantini AS (2004) Breast cancer mortality trends in two areas of province of Florence, Italy, where screening programs started in the 1970s and 1990s. Br J Cancer 90: 1780–1783 15150601 10.1038/sj.bjc.6601744 PMC2409759

[bib12] Gotzsche PC, Olsen O (2000) Is screening for breast cancer with mammography justifiable*?*. Lancet 355: 129–134 10675181 10.1016/S0140-6736(99)06065-1

[bib13] Groenendijk RB, Bult P, Noppen CM, Boetes C, Ruers TJ, Wobbes T (2003) Mitotic acticity index in interval cancers. Eur J Surg Oncol 29: 29–31 12559073 10.1053/ejso.2002.1390

[bib14] Hakama M, Elovainio L, Kajantie R, Louhivuori K (1991) Breast cancer screening as public health policy in Finland. Br J Cancer 64: 962–964 1931626 10.1038/bjc.1991.436 PMC1977451

[bib15] IARC (2002) Mammography screening can reduce deaths from breast cancer. Press Release, 139, Volume 2003. Lyon: IARC Press

[bib16] Joensuu H, Lehtimäki T, Holli K, Elomaa L, Turpeenniemi-Hujanen T, Kataja V, Anttila A, Lundin M, Isola J, Lundin J (2004) Risk for distant recurrence of breast cancer detected by mammography screening or other method. JAMA 292: 1064–1073 15339900 10.1001/jama.292.9.1064

[bib17] Klemi PJ, Parvinen I, Pylkkänen L, Kauhava L, Immonen-Räihä P, Räsänen O, Helenius H (2003) Significant improvement in breast cancer survival trough population-based mammography screening. The Breast 12: 308–313 14659145 10.1016/s0960-9776(03)00096-1

[bib18] Kricker A, Farac K, Smith D, Sweeny A, McCredie M, Armstrong BK (1999) Breast cancer in New South Wales in 1972–1992: tumor size and the impact of mammographic screening. Int J Cancer 81: 877–880 10362133 10.1002/(sici)1097-0215(19990611)81:6<877::aid-ijc7>3.0.co;2-f

[bib19] McCann J, Stockton D, Day N (1998) Breast cancer in East Anglia: the impact of the breast screening programme on stage at diagnosis. J Med Sreen 5: 42–48 10.1136/jms.5.1.42 9575460

[bib20] Paci E, Duffy SW, Giorgi D, Zappa M, Crocetti E, Bianchi S, Rosselli del Turco M (2002) Quantification of the effect of mammographic screening on fatal breast cancers: The Florence Programme 1990–96. Br J Cancer 87: 65–69 12085258 10.1038/sj.bjc.6600301 PMC2364283

[bib21] Peeters PHM, Verbeek ALM, Hendriks JHCL, Holland R, Mravunac M, Vooijs GP (1989) The occurrence of interval cancers in the Nijmegen screening programme. Br J Cancer 59: 929–932 2736229 10.1038/bjc.1989.196 PMC2246722

[bib22] Porter PL, El-Bastwissi AY, Mandelson MT, Lin MG, Khalid N, Watney AE, Cousens L, White D, Taplin S, White E (1999) Breast tumour characteristics as predictors of mammographic detection: comparision of interval- and screen detected cancers. J Natl Cancer Inst 91: 2020–2028 10580027 10.1093/jnci/91.23.2020

[bib23] Shapiro S, Coleman EA, Broeders M, Cood M, deKoonig H, Frachboud J, Moss S, Paci E, Stachenko E, Ballard-Barbash R (1998) Breast cancer screening programmes in 22 countries: current policies, administration and guidelines. Int J Epid 27: 735–742 10.1093/ije/27.5.735 9839727

[bib24] Sobin LH, Witekind C (eds) (2002) UICC TNM Classification of Malignant Tumours. 6th ed. New-York: Wiley-Liss 2002

[bib25] Tabar L, Yen MF, Vitak B, Chen H-H, Smith RT, Duffy SW (2003) Mammography service screening and mortality in breast cancer patients: 20-years follow-up before and after the introduction of screening. Lancet 361: 1405–1410 12727392 10.1016/S0140-6736(03)13143-1

